# 

**Published:** 1898-07

**Authors:** 


### Southern Medical Record

A Monthly Journal of Medicine and Surgery.

VOL. XXVIII. ATLANTA, GA., JULY, 1898. No. 7

LIBRARY SURGEON GENERAL'S OFFICE AUG.-10-1898

BERNARD WOLFF, M. D., EDITOR.

ASSOCIATE EDITORS:

A. W. GRIGGS, M. D.

J. McFADDEN GASTON, M. D.

WILLIS F. WESTMORELAND, M. D

B. M. ZETTLER, BUSINESS MANAGER.

